# Myosteatosis in Cirrhosis: A Review of Diagnosis, Pathophysiological Mechanisms and Potential Interventions

**DOI:** 10.3390/cells11071216

**Published:** 2022-04-04

**Authors:** Maryam Ebadi, Cynthia Tsien, Rahima A. Bhanji, Abha R. Dunichand-Hoedl, Elora Rider, Maryam Motamedrad, Vera C. Mazurak, Vickie Baracos, Aldo J. Montano-Loza

**Affiliations:** 1 Division of Gastroenterology & Liver Unit, University of Alberta, Edmonton, AB T6G 2X8, Canada; ebadi@ualberta.ca (M.E.); rbhanji@ualberta.ca (R.A.B.); rider@ualberta.ca (E.R.); 2 Ajmera Transplant Program, Department of Medicine, University of Toronto, Toronto, ON M5S 1A8, Canada; cynthia.tsien@gmail.com; 3 Division of Human Nutrition, University of Alberta, Edmonton, AB T6G 2P5, Canada; abha@ualberta.ca (A.R.D.-H.); motamedr@ualberta.ca (M.M.); vmazurak@ualberta.ca (V.C.M.); 4 Department of Oncology, Cross Cancer Institute, Edmonton, AB T6G 1Z2, Canada; vickie.baracos@ualberta.ca

**Keywords:** muscle quality, radiation attenuation, pathways, interventions

## Abstract

Myosteatosis, or pathological excess fat accumulation in muscle, has been widely defined as a lower mean skeletal muscle radiodensity on computed tomography (CT). It is reported in more than half of patients with cirrhosis, and preliminary studies have shown a possible association with reduced survival and increased risk of portal hypertension complications. Despite the clinical implications in cirrhosis, a standardized definition for myosteatosis has not yet been established. Currently, little data exist on the mechanisms by which excess lipid accumulates within the muscle in individuals with cirrhosis. Hyperammonemia may play an important role in the pathophysiology of myosteatosis in this setting. Insulin resistance, impaired mitochondrial oxidative phosphorylation, diminished lipid oxidation in muscle and age-related differentiation of muscle stem cells into adipocytes have been also been suggested as potential mechanisms contributing to myosteatosis. The metabolic consequence of ammonia-lowering treatments and omega-3 polyunsaturated fatty acids in reversing myosteatosis in cirrhosis remains uncertain. Factors including the population of interest, design and sample size, single/combined treatment, dosing and duration of treatment are important considerations for future trials aiming to prevent or treat myosteatosis in individuals with cirrhosis.

## 1. Introduction

Radiologically identified skeletal muscle abnormalities, including sarcopenia (low muscle mass) and myosteatosis (pathological fat accumulation in muscle), are common in patients with cirrhosis. Although extensive research over the last decade has demonstrated sarcopenia to have an independent association with poor prognosis in cirrhosis [1,2,3], little is known about the clinical implications of myosteatosis, an indicator of poor muscle quality. Muscle quality is defined by the ratio of muscle strength to mass, which is affected by changes in muscle composition [4]. In muscle, lipids can accumulate as intermuscular adipose tissue (IMAT, fat beneath the deep fascia and between adjacent muscle groups), intramuscular adipose tissue (fat between and/or within muscle fibers) and intramyocellular lipids in the form of lipid droplets [5,6,7]. Intramuscular fat may reduce muscle quality by disrupting muscle fiber alignment, thus weakening mechanical action [8].

The gold-standard technique for assessing fat infiltration into muscle is biopsy; however, limited data are available due to the invasiveness of tissue sampling. Cross-sectional imaging with computed tomography (CT) and magnetic resonance imaging (MRI) has facilitated comprehensive analysis of muscle quality. Despite the lack of ionizing radiation, MRI is expensive and time-consuming, and consistent protocols for the scanning process and software are lacking. Protocols using 3D volumetric MRI coupled with advanced image processing techniques have been recently developed, enabling assessment of muscle composition [9]. However, this technique needs to be validated and standardized in patients with cirrhosis.

In cirrhosis, CT has become a common modality for radiographic assessment of myosteatosis, mostly available as part of standard care [10]. Alteration in muscle quality by excess fat accumulation is recognized as a lower mean skeletal muscle radiodensity on CT [11]. Muscle radiodensity can be objectively assessed by CT in Hounsfield units (HU), where 0 and −1000 HU are the radiodensity of distilled water and air at standard pressure and temperature [12], respectively. The predefined CT radiodensity threshold value for demarcating muscle is in the range of −29 to 150 HU [13]. Fat infiltration into the muscle reduces the radiodensity measured in HU on CT; however, controversies remain regarding the definition of low-radiodensity muscle, as ranges from 0 to 29 HU and −29 to 30 HU have been applied as low-radiodensity muscle in previous studies [11].

Myosteatosis may not necessarily occur at the same time as the loss of muscle mass. It remains unclear whether pathological fat accumulation in muscle results from the loss of muscle mass or whether it occurs prior to alterations in muscle mass [14]. Although myosteatosis may be a feature of muscle loss, data regarding various interactions between myosteatosis and sarcopenia are still controversial. Myosteatosis was reported in 93% of sarcopenic patients with chronic liver disease [15], but no interaction effect of sarcopenia and myosteatosis on cirrhosis complications was reported [16].

Despite the significant clinical implications of myosteatosis in cirrhosis, a standardized definition for myosteatosis and the exact mechanisms associated with its development have not been thoroughly characterized. The elucidation of abnormalities in muscle physiology and underlying molecular mechanisms is essential in developing reversal agents. Therefore, the goal of this review is to summarize important results from the literature regarding the diagnosis of myosteatosis, pathogenic mechanisms and potential therapeutic targets. We will also outline important considerations for the design of future clinical trials in cirrhosis.

## 2. Search Strategy

A literature search was performed in MEDLINE (OvidSP) until October 2021 using the subject heading terms “myosteatosis”, “muscle radiodensity”, “muscle attenuation”, “muscle fat” and “intramuscular fat” pooled with the Boolean operator “AND” to the search terms “cirrhosis”, “chronic liver disease”, “end-stage liver disease” and “liver transplant”. The search was restricted to full-text papers published in English. A manual review of the literature was conducted to include papers discussing diagnosis, outcomes, pathophysiological mechanisms and potential interventions.

**Figure 1:**
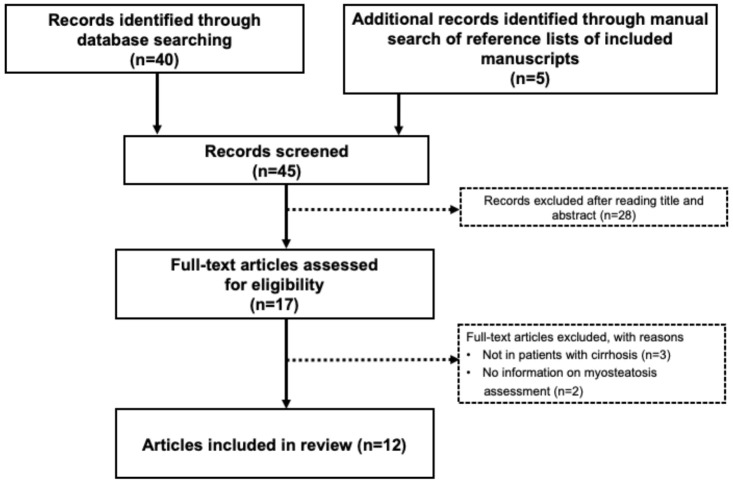
Flow chart of literature search and inclusion of studies in the review.

The preliminary search yielded 45 potentially relevant articles. After screening titles and abstracts, 28 papers were excluded, and therefore, 17 full-text articles were reviewed. Of those 17 articles, five were excluded because they did not include patients with cirrhosis or because no explanation for myosteatosis determination was provided. Figure 1 presents a detailed flow chart of the selection of the 12 studies included in this review. Study eligibility was assessed independently by M.E. and A.M.L., and any inconsistencies were resolved by a consensus of the reviewers. Relevant articles are discussed below.

## 3. Diagnosing Myosteatosis

**Figure 2:**
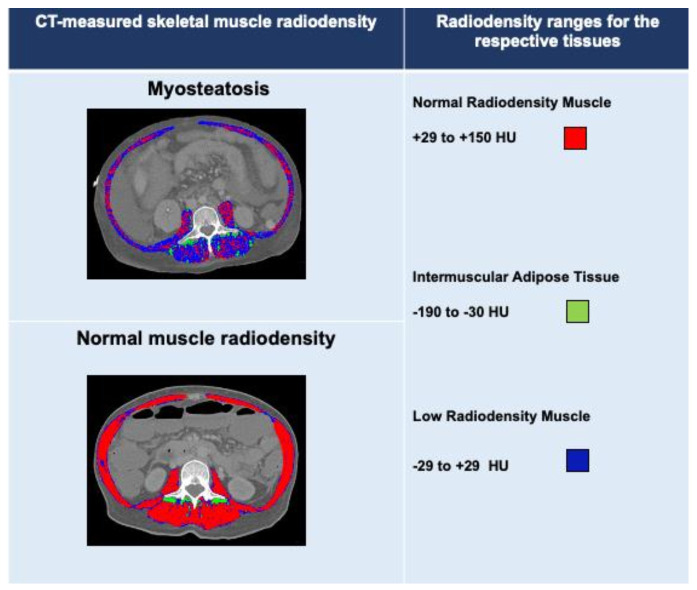
Abdominal computed tomography images taken at the third lumbar vertebra to quantify IMAT and muscle radiodensity in two patients with cirrhosis.

Optimal cutoff values to define normal and low-radiodensity muscle in relation to adverse outcomes have not been established in patients with cirrhosis. Myosteatosis in cirrhosis has been defined using IMAT cross-sectional areas or mean muscle radiodensity. IMAT represents, in particular, intermuscular adipose tissue areas, whereas the low mean muscle radiodensity represents poor-quality muscle with areas containing intramuscular adipose tissue and intramyocellular lipids [17]. Figure 2 illustrates the IMAT cross-sectional areas and total skeletal muscle radiodensity estimation at L3 in two patients with cirrhosis. Mean skeletal muscle radiodensity was 19 HU in a patient with myosteatosis and 48 HU in a patient with normal muscle radiodensity. Areas composed of low-radiodensity muscle (<29 HU) are predominant in a patients with myosteatosis, whereas areas of normal-radiodensity muscle (≥29 HU) are prevalent in patients without myosteatosis. The IMAT cross-sectional area was 4 cm^2^/m^2^ and 6 cm^2^/m^2^ in a patient with and without myosteatosis, respectively.

Radiodensity ranges used for the analysis of normal radiodensity (red), low-radiodensity (dark blue) muscle and intermuscular adipose tissue (IMAT; green) are shown. Mean skeletal muscle radiodensity was 19 HU in a patient with myosteatosis and 48 HU in a patient with normal muscle radiodensity or no myosteatosis. Areas composed of low-radiodensity muscle (<29 HU) are predominant in patients with myosteatosis, whereas areas of normal-radiodensity muscle (≥29 HU) are prevalent in patients with myosteatosis. The IMAT cross-sectional area was 4 cm^2^/m^2^ and 6 cm^2^/m^2^ in a patient with and without myosteatosis, respectively.

Myosteatosis has been widely diagnosed based on the mean radiodensity (HU) value of the entire or partial muscle cross-sectional areas on CT. Values derived from cancer populations have been commonly applied to predict clinical outcomes in patients with cirrhosis. A mean third lumbar vertebra (L3) muscle radiodensity cutoff of <33 HU in patients with a BMI ≥25 kg/m^2^ and <41 HU in those with a BMI <25 kg/m^2^ was established in cancer patients to predict mortality [18]. Using these cutoffs for myosteatosis, 52% of patients with cirrhosis met criteria for myosteatosis [19]. Given fluid retention in a majority of patients with cirrhosis, the applicability of these BMI-dependent cutoffs is questionable. Moreover, the higher lipid storage capacity of skeletal muscle in females compared to males [20] requires sex-specific cutoffs for myosteatosis to be defined.

In patients with cirrhosis, the optimal cutoff to predict 12-month mortality was determined using the psoas muscle radiodensity at the level of the fourth to fifth vertebra. Psoas muscle radiodensity below 43.14 HU was associated with higher 12-month mortality after adjusting for age, sex and Child–Pugh score [21]. When the predictive performance of psoas muscle radiodensity in predicting short- and long-term outcomes after deceased donor LT was compared to the performance of mean radiodensity of L3 skeletal muscle, better performance was observed using the latter in predicting post-LT mortality [22]. However, no sex-specific cutoffs using the mean radiodensity of L3-skeletal muscle have been established in patients with cirrhosis to predict pre-liver transplant (LT) mortality. Limited predictive accuracy of psoas muscle compared to whole muscle at L3 has been reported in other studies [23], highlighting the importance of establishing optimal cutoffs in diagnosing myosteatosis.

Myosteatosis has also been defined based on IMAT-normalized radiodensity or cross-sectional areas [24,25]. CT radiodensity of the multifidus muscles (HU) was divided by the radiodensity of subcutaneous adipose tissue to determine IMAT. L3-IMAT values >−0.44 in male and >−0.37 in female patients with cirrhosis have been used as cutoffs to delineate myosteatosis [24]. To determine IMAT, predefined radiodensity ranges of −190 to −30 HU [26] were applied to identify the cross-sectional areas of IMAT within the L3 muscle areas [25].

Assessment of myosteatosis in previous studies was performed using CT image analysis. MRI also has the capability of measuring fat infiltration into organs and muscles. Recently, MRI-derived fat fraction of erector spinae muscles was measured at the point of the highest muscle volume, with myosteatosis established as a fat fraction below 0.8 in LT recipients [27].

## 4. Clinical Significance of Myosteatosis

Reduced muscle radiodensity negatively correlates with clinical outcomes (Table 1). It is an independent predictor of increased pre-LT mortality in cirrhosis (adjusted for severity of the liver disease) and may be related to deterioration in physical conditioning [19,25]. Myosteatosis has also been shown to be associated with complications of cirrhosis, such as hepatic encephalopathy (HE), with a prevalence of 70% in patients with overt HE, as compared to 45% in patients without HE [16]. Independent of sarcopenia, myosteatosis is associated with the presence of minimal HE and the development of overt HE in patients with cirrhosis [28]. Significant improvement in CT-measured muscle radiodensity following transjugular intrahepatic portosystemic shunt was reported in previous studies [29,30].

When LT is a competing event, myosteatosis, sarcopenia, MELD and HE are independently associated with mortality in cirrhotic patients evaluated for LT. In an attempt to improve prognostication, the MELD–Sarco–Myo–HE score was developed, which improved MELD accuracy in predicting 3- and 6-month mortality. By removing myosteatosis from the score, a significant loss in accuracy in comparison to the MELD–Sarco–Myo score was noticed, suggesting the clinical importance of myosteatosis in predicting pre-LT mortality [31].

When myosteatosis was defined based on IMAT-normalized radiodensity, it was associated with multidimensional frailty in hospitalized male patients with cirrhosis but not in female patients. A correlation between IMAT and frailty index (*r*  =  0.238, *p* =  0.018) and higher frequency of myosteatosis was only observed in frail male patients (62.5 vs. 15.8%, *p* =  0.001) compared to the non-frail group [24]. In another study, mean muscle radiodensity <34 HU was associated with higher risk of waitlist mortality (HR 8.88, 95% CI 1.95–40.41, *p* = 0.005), whereas no association between IMAT cross-sectional areas and waitlist mortality was confirmed in multivariate sensitivity analysis of 261 patients with cirrhosis listed for LT. It was speculated that CT-determined IMAT might be less accurate than mean muscle radiodensity because it constitutes only a small portion of CT images and therefore is subject to interobserver bias [25].

Preoperative myosteatosis, assessed using the fat fraction of MRI, in patients who underwent LT was associated with increased length of hospital stay post-LT. There was also a trend toward higher risk of graft loss (adjusted HR, 2.07; 95% CI, 0.92–4.64; *p* = 0.08) and mortality (adjusted HR 2.24, 95% CI, 0.93–5.41; *p* = 0.07) [27]. Without adjusting for MELD, sex, race, BMI and weight at LT, and donor and recipient age and etiology, the association between myosteatosis and graft survival was significant (HR 2.08, 95% CI 1.04–4.15, *p* = 0.037). However, given the small number of outcomes in this study, there remains a possibility of multivariate model overfitting.

The impact of low skeletal muscle radiodensity on perioperative outcomes remains controversial. In patients receiving deceased donor orthotopic LT, mortality and complication rates over the first 3 months, length of intensive care unit (ICU) and hospital stay, and procedural costs were higher in patients with myosteatosis. There were no differences in long-term graft and patient survival between groups [32,33], suggesting myosteatosis is a key factor in predicting short-term outcomes following LT [22]. In another study, despite longer ICU stay in patients with cirrhosis and myosteatosis, no difference in the hospital length of stay or bacterial infections was seen in the first 90 days post-LT between the groups [19]. Inclusion of myosteatosis within the balance-of-risk (BAR) score, a well-established score for identification of high-risk recipients and/or donor–recipient combinations, improved outcome prediction following orthotopic LT, indicating the possible further importance of myosteatosis as a predictor of immediate outcomes [32].

**Table 1:** Summary of studies investigating clinical significance of myosteatosis in cirrhosis.

Author/ Year	Study Population	Cutoff for Myosteatosis	Prevalence of Myosteatosis	Outcome Associated with Myosteatosis
Montano-Loza et al., 2016 [19]	678 patients with cirrhosis evaluated for LT	L3 muscle radiodensity <41 HU in patients with a BMI up to 24.9 and <33 in those with a BMI ≥25 kg/m^2^	52%	Myosteatosis was an independent predictor of long-term mortality.
Bhanji et al., 2018 [16]	675 patients with cirrhosis evaluated for LT	L3 muscle radiodensity <41 HU in patients with a BMI up to 24.9 and <33 in those with a BMI ≥25 kg/m^2^	52%	Myosteatosis was identified in 70% of patients with overt hepatic encephalopathy and was an independent predictor of both hepatic encephalopathy and mortality.
Kalafateli et al., 2018 [21]	98 consecutive patients with cirrhosis	Average psoas muscle radiodensity at the level of the fourth to fifth lumbar vertebrae below 43.14 HU	20%	Myosteatosis was associated with a higher risk of 12-month mortality after adjusting for age, sex and Child–Pugh score.
Tachi et al., 2018 [15]	362 patients with chronic liver disease	L3 muscle radiodensity <41 HU in patients with a BMI up to 24.9 and <33 in those with a BMI ≥25 kg/m^2^	82%	Myosteatosis, low BMI, low alanine aminotransferase and female sex were predictors of sarcopenia in patients with chronic liver disease.
Nardelli et al., 2019 [28]	64 patients with cirrhosis who were administered a test to detectminimal hepatic encephalopathy	L3 muscle radiodensity <41 HU in patients with a BMI up to 24.9 and <33 in those with a BMI ≥25 kg/m^2^	38%	Myosteatosis was associated with the presence of minimal hepatic encephalopathy and the development of overt hepatic encephalopathy.
Lattanzi et al., 2019 [31]	249 patients with cirrhosis evaluated for LT	L3 muscle radiodensity <41 HU in patients with a BMI up to 24.9 and <33 in those with a BMI ≥25 kg/m^2^	54%	MELD–Sarco–Myo–HE score was developed, which improved MELD accuracy in predicting 3- and 6-month mortality.
Czigany et al., 2020 [32]	225 consecutive recipients of orthotopic LT	L3 muscle radiodensity <41 HU in patients with a BMI up to 24.9 and <33 in those with a BMI ≥25 kg/m^2^	44%	Higher mortality and complication rates over the first 3 months, length of intensive care unit and hospital stay and procedural costs in patients with myosteatosis, with no differences in long-term graft and patient survival between groups.
Shenvi et al., 2020 [27]	180 recipients of LT	Preoperative fat fraction of MRI <0.8	16%	Myosteatosis was associated with increased length of hospital stay post-LT.
Meister et al., 2021 [22]	264 consecutive recipients who underwent deceased donor orthotopic LT	L3 muscle radiodensity <26.6 HU in female and <28.6 HU in male patients	25%	Applied cutoffs identified patients at risk for inferior short- but not long-term graft and patient outcomes after LT.
Bot et al., 2021 [25]	261 patients listed for LT	Lowest quartile of muscle radiodensity at the level of L3 (<34.0 HU)	25%	Higher risk of waitlist mortality in patients with myosteatosis (HR of 9.12 (HR 8.88, 95% CI: 1.95–40.41, *p* = 0.005). No association between intramuscular adipose tissue content and wait-list mortality in multivariate sensitivity analysis.
Feng et al., 2021 [24]	202 hospitalized patients with cirrhosis	L3 radiodensity of the multifidus muscles normalized to the radiodensity of subcutaneous adipose tissue (>−0.44 in male and >−0.37 in female patients)	19%	Higher incidence of myosteatosis in frail male patient (62.5 vs. 15.8%, *p* = 0.001).
Irwin et al., 2021 [34]	106 LT recipients	L3 muscle radiodensity <41 HU in patients with a BMI up to 24.9 and <33 in those with a BMI ≥25 kg/m^2^	72%	Myosteatosis was associated with a higher risk of post-LT adverse outcomes, including mortality and allograft failure at 1 year, as well as longer hospital and intensive care unit stays.

Abbreviations: BMI, body mass index; HU, Hounsfield unit; L3, third lumbar vertebra; LT, liver transplantation.

In 106 LT recipients, myosteatosis was associated with higher risk of post-LT adverse outcomes, including mortality at 1 year (HR, 3.3; 95% CI, 1.00–11.13; *p* = 0.049), allograft failure (HR, 4.1; 95% CI, 1.2–13.5; *p* = 0.021) and longer hospital and intensive care unit stays. Myosteatosis was determined using unenhanced abdominal CT images taken 6 months before or 1 month post-LT [34]. Given the catabolic stress of LT, potential discrepancies between applying pre- and post-LT CTs in determining body composition features have not been clarified.

## 5. Mechanisms of Myosteatosis in Cirrhosis

Emerging evidence suggests the preferential storage of extramyocellular lipids in muscles with a dietary lipid overload of short duration [35]. During the early phase of lipid overload, oxidative muscles may resist intramyocellular lipid accumulation through augmented β-oxidation capacity; however, this capability may fluctuate depending on muscle type [35]. Following excess exposure to fatty acids, oxidative muscles dispose free fatty acids by oxidation, whereas re-esterification of free fatty acids to triglycerides occurs in glycolytic muscles due to the lower mitochondrial oxidative phosphorylation [36,37]. This suggests pathophysiological adaptation to lipotoxicity differs by muscle type. Therefore, differences between muscle types and sex-dependent differences in muscle metabolism are important considerations. In general, higher levels of lipids accumulate in oxidative rather than glycolytic muscles, and lipid oxidation is the favored energy source in oxidative muscles. This demonstrates muscle-specific responses in intramyocellular fatty acid metabolism [38].

Excessive fat accumulation in muscle may impact muscle fiber orientation and is associated with muscle inflammation, reduced muscle strength and physical performance [39,40]. An early adaptation response to this lipotoxicity is a transition of muscle fibers from fast to slow (type II to type I), which leads to enhanced muscle oxidative capacity [38]. Chronic adaptation response may be associated with higher amounts of glycolytic metabolites concurrent with a reduction in mitochondrial lipid oxidation. This indicates that muscle fibers are using lower levels of lipids for oxidation in myosteatosis [5]. Impaired muscle oxidative capacity in turn triggers muscle fiber atrophy. Fiber-type-specific lipid accumulation has not yet been defined in cirrhosis.

In vitro and in vivo experimental models have been used to explore the mechanisms of myosteatosis. Animal studies of myosteatosis are mainly diet-induced obesity models with increased intramyocellular lipid accumulation and decreased oxidation of lipids in muscle fibers. However, a muscle-specific pattern was reported for high-fat-induced myosteatosis [41]. Culturing human muscle cells isolated from a vastus lateralis muscle biopsy with human obese subcutaneous adipose-tissue-conditioned medium impaired myogenesis and promoted intramyocellular lipid accumulation in myotubes [42]. Using C2C12 cells, a recognized in vitro model of skeletal muscle cells, a reverse association between skeletal muscle lipid accumulation and AMPK (AMP-activated protein kinase) activity was found [43]. In palmitate-treated C2C12 myotubes, leucine reduced lipid accumulation through regulation of mitochondrial function in an mTORC1-independent manner. Palmitate-treated C2C12 myotubes have been used to imitate in vivo lipid accumulation in obese skeletal muscle [44].

**Figure 3:**
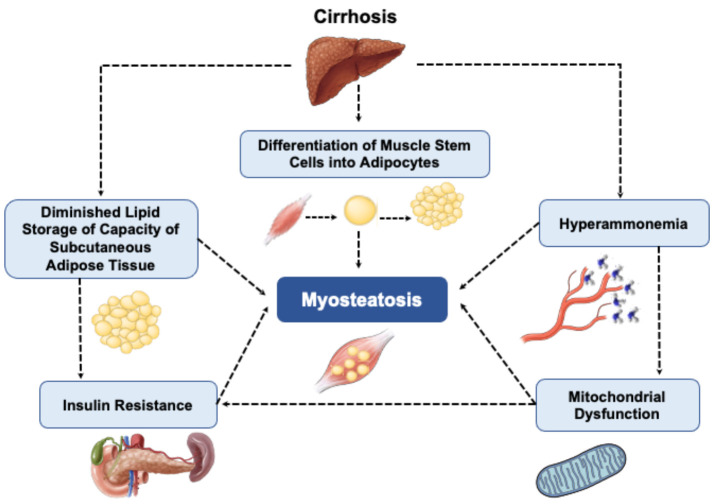
Summary of pathways contributing to myosteatosis in cirrhosis.

Data regarding the mechanisms by which excess lipid accumulates within muscle in cirrhosis is sparce, but it might be related to the metabolic abnormalities associated with liver failure (Figure 3). Hyperammonemia may play an important role in the pathophysiology of myosteatosis in cirrhosis. Increased skeletal muscle ammonia uptake induces skeletal muscle mitochondrial dysfunction via cataplerosis of α-ketoglutarate [45], which subsequently results in impaired mitochondrial oxidative phosphorylation and diminished lipid oxidation in muscle [46]. In an experimental model of myosteatosis, increased lipid storage in supraspinatus muscle of Sprague–Dawley rats was associated with a reduction in the expression of genes involved in the uptake of fatty acids, transportation and β-oxidation within the mitochondria. These changes probably lead to lipid-induced inflammation and increased reactive oxygen species (ROS) generation in the muscle [5].

Hyperammonemia, insulin resistance, mitochondrial dysfunction, diminished lipid storage capacity of subcutaneous adipose tissue and age-related differentiation of muscle stem cells into adipocytes have been also suggested as potential mechanisms contributing to myosteatosis.

Mitochondrial dysfunction has been suggested as a putative contributor to skeletal muscle insulin resistance [47], as the accumulation of triacylglycerol and lipid molecules, such as diacylglycerol and ceramide, interrupts GLUT-4 translocation and triggers insulin resistance [48]. Considering the importance of mitochondrial oxidative phosphorylation in ATP production in skeletal muscle, mitochondrial dysfunction and low cellular levels of ATP in myosteatosis may impair protein synthesis via reduced insulin-stimulated ATP synthesis, ultimately leading to reduced muscle mass [47]. Whether reversing myosteatosis by improving mitochondrial function is accompanied by improved muscle mass requires further investigation.

Myosteatosis was the first muscle alteration identified in both early and fibrosing preclinical models of non-alcoholic steatohepatitis (NASH). The degree of fat infiltration into muscle was correlated with the severity of liver disease and inflammation rather than insulin resistance or visceral fat accumulation [49]. In line with this finding, a reduction in myosteatosis degree was associated with a reduction in liver stiffness, independent of weight loss, in 48 obese patients with metabolic-dysfunction-associated fatty liver disease (MAFLD) [50]. Lipotoxicity associated with myosteatosis impacts muscle secretome, which may cause subsequent alterations in muscle mass and function [51].

Although obesity and insulin resistance are two main conditions associated with myosteatosis, insufficient storage of lipids in subcutaneous adipose tissue has also been recognized as a potential contributor to myosteatosis [6,42]. A decreased ability of subcutaneous adipose tissue to store lipids and ectopic fat accumulation in other locations, such as visceral adipose tissue, liver and muscle, is linked with inflammation and insulin resistance [52]. Excessive lipid availability and flux into muscle are determinant factors in skeletal muscle lipid deposition and accumulation of lipotoxic intermediates [53]. Improving lipid storage capacity of subcutaneous adipose tissue by proliferator-activated receptor gamma (PPAR-gamma) agonist enhanced insulin sensitivity [54]. Therefore, improved storability of adipose tissue and elevated muscle fatty acid oxidation can contribute to lower circulating lipid levels and consequently lower lipotoxicity [55]. Whether myosteatosis in cirrhosis is associated with metabolic disorders associated with high adiposity, or impaired storage of lipids in subcutaneous adipose tissue remains unknown. Lastly, age-related differentiation of muscle stem cells into adipocytes [56] has also been suggested as a potential mechanism contributing to myosteatosis.

Results of studies investigating the mechanisms underlying myosteatosis in cirrhosis should be interpreted with caution, as the majority of data on lipid accumulation in muscle comes from experimental studies (in males) in which the impact of high-fat diet or skeletal muscle injury on lipid accumulation in muscle was investigated. Although animal models are required to promote our understanding of myosteatosis in cirrhosis, they may not necessarily represent the clinical course of cirrhosis. Therefore, studies assessing cirrhosis-associated myosteatosis are needed.

## 6. Potential Pharmaceutical Targets Based on Pathogenic Pathways

Nutritional and pharmacological interventions that influence ammonia metabolism have attracted interest to improve myosteatosis in cirrhosis. Other agents that deserve future investigation are the long-chain n-3 polyunsaturated fatty acids (PUFAs), which may improve mitochondrial oxidative phosphorylation capacity. Determining the efficacy of these agents will require a careful definition of endpoints and detailed information on patients’ actual physical activity level and nutritional intake.

### 6.1. Ammonia-Lowering Treatments

Excessive amounts of ammonia delivered to muscle are now well recognized as the key multipotent metabolic contributor to loss of muscle quantity and quality in cirrhosis. However, data exclusively assessing the impact of ammonia-lowering strategies on myosteatosis in patients with cirrhosis are lacking. Agents used to lower ammonia levels and improve mitochondrial function may have a beneficial role in myosteatosis treatment or prevention. A reduction in plasma ammonia levels can be achieved using long-term therapeutic nutritional supplementation with branched-chain amino acid (BCAA) mixtures [57]; L-ornithine L-aspartate mixture with rifaximin [58]; or leucine, which increases mitochondrial oxidation in hyperammonemic states [59]. Improved mitochondrial function and reduced ammonia levels have also been observed with the use of l-carnitine in a dose-dependent manner in patients with cirrhosis [60]. L-ornithine and L-aspartate, in combination with rifaximin, decreased ammonia levels in plasma and muscle of an experimental model of hyperammonemia and led to improved muscle protein synthesis and function. A significant increase in type II fiber size and reduction in the expression of myostatin and autophagy markers was noticed using these ammonia-lowering agents [58]. Considering the contribution of hyperammonemia in the pathogenesis of myosteatosis in cirrhosis, the potential ability of L-ornithine and L-aspartate to effectively lower blood ammonia and subsequently improve myosteatosis requires further investigation.

### 6.2. Long-Chain n-3 Polyunsaturated Fatty Acids

Evidence suggests that omega-3 PUFAs improve mitochondrial oxidative phosphorylation capacity in human skeletal muscle and therefore may be pivotal in myosteatosis treatment [61]. The ability of eicosapentaenoic acid (EPA) and docosahexaenoic acid (DHA) to prevent tumor-associated myosteatosis was found in a preclinical model of colon cancer [62]. The metabolic consequence of omega-3 polyunsaturated fatty acids in reversing myosteatosis in cirrhosis remains to be investigated.

## 7. Considerations for Future Research Trial Designs

Myosteatosis is an endpoint in trials aiming to improve skeletal muscle quality. When designing an intervention that targets a specific underlying mechanism, maximizing potential benefit and minimizing possible toxicity are important determinants of trial efficacy [14]. Practical considerations for the implementation of clinical trials include the population of interest, design and sample size, single/combined treatment, duration and dosing of the intervention and treatment endpoints.

Preventive or treatment trials for skeletal muscle abnormalities require a valid assessment of the abnormality. The diagnosis of myosteatosis for admission of patients into clinical trials and the effective measurement of myosteatosis reversal overtime should be established based on CT-measured muscle radiodensity [3]. CT may be the only option in trials assessing the quality of muscle by measuring muscle radiodensity. Besides reliability and sensitivity of modalities to capture changes in muscle radiodensity, cost, availability and feasibility of appropriate techniques to quantify changes should be considered, particularly in large or longitudinal studies.

The population of interest is another important consideration in order to identify the group that may benefit the most from the intervention. Patients with mild-to-moderate-severity myosteatosis may be the most responsive to therapy. The lack of myosteatosis stages in cirrhosis makes it difficult to identify the best target population and generalize the findings, as the preventative approach in early-stage patients might be different than treatment strategies in patients with moderate-to-severe myosteatosis.

For trials assessing the impact of pharmacological interventions on muscle quality, complementary therapies such as exercise or nutritional support might result in differences between responders and non-responders, and therefore, detailed information on these therapies needs to be accounted for. The duration of intervention should be long enough to ensure that the magnitude of change in the treatment endpoint is of adequate length and that the outcome is not just due to normal variation. The accepted change for muscle cross-sectional area on CT is a difference greater than 2%, and any change between −2 and +2% is not clinically meaningful and may reflect tissue maintenance [63]. However, such a threshold has not been identified for changes in muscle radiodensity.

Factors such as treatment endpoint, population of interest, annual rate of muscle change and severity of liver diseases are important considerations to identify the length of the trial and should be taken into account in designing trials in cirrhosis [14,64]. Compliance with intervention is a significant determinant of clinical trial outcomes. Poor patient adherence is a frequently described drawback of clinical trials, which could be improved through optimal design, dosing and the appropriate type of supplement [65]. Lastly, features such as time point of the disease’s trajectory, sex, age, race and medications may also act as confounding variables, and therefore, a well-adjusted distribution using design and analytic strategies should include a homogeneous population, especially in small trials [14]. The importance of sample size calculation to determine the optimal number of patients to detect a clinically meaningful difference is another consideration for designing trials [66]. Employing the aforementioned considerations in future large trials investigating both prevention and treatment of skeletal muscle abnormalities in patients with cirrhosis may ensure positive outcomes.

## 8. Conclusions

Among cross-sectional imaging techniques to diagnose myosteatosis in patients with cirrhosis, abdominal CT constitutes the most studied technique. Myosteatosis is mainly defined as a low muscle radiodensity on CT. It is associated with a poor prognosis, including mortality, and complications such as HE in cirrhosis. Although myosteatosis confers prognostic value in cirrhosis, it is not included in conventional scores for prognosis, such as the MELD or Child–Pugh scores. This may need to be evaluated in future studies. Identifying the efficacy of a potential intervention for myosteatosis necessitates an accurate validated definition, which is currently lacking in cirrhosis. Standard modalities and definitions of myosteatosis (including sex-based cutoffs) in cirrhosis should be established and validated for use in clinical practice. It is possible that myosteatosis may also contribute to sarcopenia. Future research should also investigate the impact of the concurrent presence of muscle abnormalities, i.e., sarcopenia and myosteatosis, in such patients. The use of non-contrast versus contrast-enhanced CT scans should be reported in studies of myosteatosis, given the higher muscle radiodensity in the arterial and portal venous phase compared to non-contrast-phase CTs [67]. Although myosteatosis has been defined as pathological lipid accumulation in muscle, the composition of lipids seems to play an important role in the pathogenesis of myosteatosis rather than the total amount of lipids per se [11]. Future studies need to investigate an association between the composition of lipids stored in muscle and the presence of myosteatosis. Although myosteatosis is associated with worse outcomes in cirrhosis, no standard treatments are available. A better understanding of the mechanisms underlying myosteatosis is key to planning clinical trials with the aim of reversing this skeletal muscle abnormality. In summary, this review emphasizes the need for prospective studies with a larger number of patients to develop our existing knowledge of the predictive value of myosteatosis in cirrhosis and trial new treatments.

## Data Availability

Not applicable.
